# Looking back: tracing the origins of sleep across the animal kingdom

**DOI:** 10.3389/fnins.2026.1886828

**Published:** 2026-08-18

**Authors:** Jimmy J. Fraigne, Zoltan A. Torontali, Jessica C. Pressey

**Affiliations:** Department of Cell & Systems Biology, University of Toronto, Toronto, ON, Canada

**Keywords:** circadian rhythm (CR), comparative & evolutionary neuroscience, sleep evolution, sleep wake, temporal niche

## Abstract

The evolutionary purpose of sleep is an enduring biological mystery of the animal kingdom. Most research to date has investigated the processes that occur during sleep as opposed to why it evolved in the first place, and a recent theory proposes this behavior evolved to restrict activity to one temporal niche. Circadian rhythms are intimately linked to sleep, and in derived species, sleep-wake behaviors have been well characterized and are regulated by neuronal circuits. However, recent studies suggest sleep is also present in species lacking a centralized nervous system, and in this review, we discuss the interplay between ancient circadian processes and their potential role in the evolution of sleep behavior. By approaching the evolution of sleep using a comparative physiology perspective, we present evidence that layers of molecular oscillators and highly conserved sleep signaling molecules predate neurons, and sleep is a fundamentally ancient process.

## Introduction

Sleep is one of the most enigmatic behaviors observed across the animal kingdom, and is essential for animal survival (Montagna and Lugaresi, 2002; Rechtschaffen and Bergmann, 2002; Shaw et al., 2002; Stephenson et al., 2007). This biological process is fundamental for maintaining adaptive physiological energy balance within an animal (Siegel, 2009, 2022), and can be characterized using the following behavioral criteria: (1) behavioral quiescence; (2) reversible state; (3) species-typical posture; (4) reduced responsiveness to stimuli; and (5) a homeostatic regulation of the quiescent state (Campbell and Tobler, 1984). Research has focused on the mechanisms that explain what happens during sleep, and sleep pressure is driven by factors such as metabolic homeostasis, synaptic homeostasis, DNA repair and energy allocation (Xie et al., 2013; Schmidt, 2014; Tononi and Cirelli, 2014; Zada et al., 2019). While these mechanisms provide support for why sleep must happen, they do not explain the reasons as to why sleep evolved.

In 2020, Freiberg published an opinion piece that extends earlier conclusions made by Siegel (2005, 2009, 2022), proposing that the evolutionary origin of sleep is driven by the need to specialize to one temporal niche. As day and night represent two temporal niches, it is not optimal to specialize to both. By sleeping, animals “change their physiology each day” and limit behaviors to one niche (Freiberg, 2020). Many organisms across phyla demonstrate sleep behaviors despite differences in nervous system architecture, and this might include animals which do not develop neurons. While many studies have focused on mammalian species, this review examines comparative studies of brain circuits in bilaterians and explores whether a common sleep regulatory framework exists. We discuss evidence that sleep is a fundamentally ancient process that might have evolved before a centralized nervous system (CNS) and consider the question: Are neural circuits required for sleep?

## The link between sleep and circadian rhythms

Circadian rhythms are endogenous ∼24-h oscillations of physiological processes that persist in the absence of environmental cues and critically gate the timing of sleep. Considerable work shows circadian rhythms rely on a well-defined molecular mechanism called the transcription-translation feedback loop (TTFL) (for detailed review see Takahashi, 2017). The original discovery of the *Period* gene was made in the invertebrate model *Drosophila melanogaster* (Konopka and Benzer, 1971), and the rhythmic expression of Period (PER) is essential for maintaining the timing of the clock. This elegant feedback circuit is cell-autonomous, and all vertebrates express homologous core TTFL genes. Environmental cues (zeitgebers) entrain this rhythm to synchronize with the environment and optimize energy balance, reproduction, immunity and survival with the daily light cycle (Ouyang et al., 1998; Panda, 2016; Scheiermann et al., 2018).

Circadian rhythms essentially restrict the timing of sleep to one temporal niche, and with increased organismal complexity, additional layers of regulation were incorporated to orchestrate the onset and maintenance of sleep-wake behaviors at the correct time of day. Below, we review the core structures regulating vertebrate sleep-wake cycles and discuss analogous circuits in invertebrates.

## The framework of sleep-wake neural circuits is conserved across species

**FIGURE 1:**
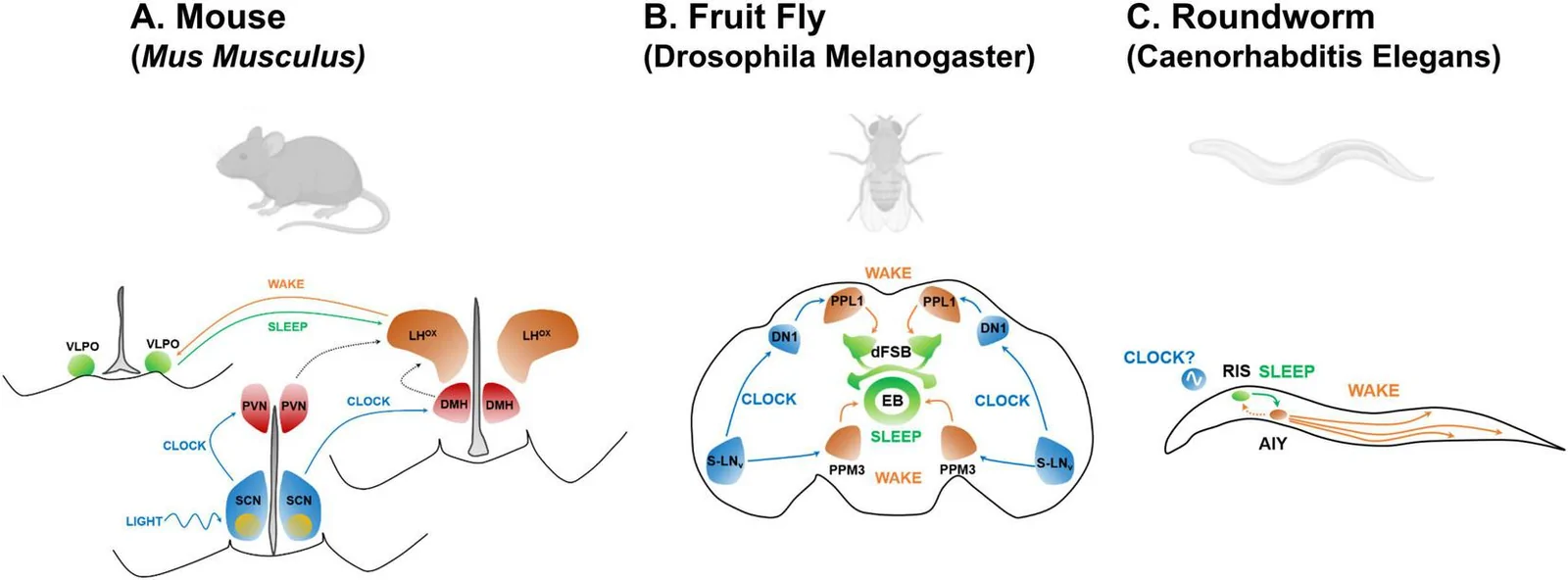
Sleep-wake and circadian circuits in vertebrates and invertebrates. The framework for the regulation of sleep-wake regulation by a bi-stable distributed circuit for state switching driven by a circadian clock is conserved across species. **(A)** The sleep-wake and circadian circuits of vertebrates have been well studied, especially in mammals. The schematic represents a small portion of the neural network described in mice (*Mus musculus*). Light via the retinohypothalamic tract (RHT) activates the suprachiasmatic nucleus (SCN, the circadian master clock, blue). The SCN modulate the activity of sleep- and wake-promoting nuclei via relay (red region) in the paraventricular nucleus (PVN) and dorsomedial hypothalamus (DMH). Modulation of the activity of these downstream targets of the SCN explains the diversity in the control of sleep timing in diurnal vs. nocturnal species. Wake-promoting orexin neurons of the lateral hypothalamus (LH, orange region) silence sleep-promoting galanin neurons of the ventrolateral pre-optic area (VLPO, green region), which in turn silence orexin neurons of the LH. **(B)** In the fruit fly (*Drosophila melanogaster*), sleep-promoting cholinergic and glutamatergic neurons of the dorsal fan-shaped body (dFSB) and the ellipsoid body (EB) gate downstream locomotion and sensory circuits. Wake-promoting dopamine neurons of the protocerebral posterior lateral 1 (PPL1) and the protocerebral posterior medial 3 (PPM3) modulate the activity of the dFSB and EB to promote arousal. Their activity is regulated by circadian neurons of the small ventrolateral neurons (s-LNv) and the dorsal neurons 1 (DN1). **(C)** In the roundworm (*Caenorhabditis elegans*), a single sleep-promoting RIS neuron releases GABA and FLP-11 to inhibit motor and sensory circuits in part through silencing of the AIY neurons. A circadian rhythm cellular mechanism regulates the activity of these neurons. Created in part with BioRender. Pressey, J. (2026) https://BioRender.com/6yskwdb, and in part with FigureLabs (https://chat.figurelabs.ai/verify/FL-PUB-20260731-YKU8BO).

The timing of sleep is shaped by the interplay between environmental conditions, the homeostatic need for sleep and the physiologic functions gated by the circadian pacemaker. Despite a broad diversity of temporal niches, the neural circuits governing the transitions between sleep-wake states share a remarkably conserved core architecture. Animals rapidly transition between sleep and wake, and the smooth transition in and out of sleep is explained by the reciprocal anatomical connections of sleep and wake-promoting nuclei (Saper et al., 2001). Wakefulness is initiated by the ascending reticular activating system (ARAS), which is composed of a distributed network of neurons located in the brainstem and hypothalamus. Wake-promoting regions include (1) noradrenergic neurons of the locus coeruleus (LC), (2) histaminergic neurons of the tuberomammillary nucleus (TMN), (3) serotonergic neurons of the dorsal raphe nucleus (DRN), (4) dopaminergic neurons of the midbrain, (5) cholinergic neurons of the pedunculopontine and laterodorsal tegmental nuclei (PPT/LDT), and (6) orexinergic neurons of the lateral hypothalamus (LH) (for a comprehensive review see Sulaman et al., 2023). These ascending arousal structures inhibit, via interneurons, the dominant sleep-promoting and galanin-containing neurons of the ventrolateral preoptic area (VLPO) (Sherin et al., 1996, 1998; Steininger et al., 2001). Reciprocally, the activity of wake-promoting neurons is suppressed during sleep by the VLPO (Figure 1A), a pattern documented across nocturnal and diurnal mammals and avian species alike (Sherin et al., 1996, 1998; Steininger et al., 2001). Galanin-containing neurons are also present in the pre-optic area of zebrafish, highlighting the conservation of this necessary inhibitory structure from teleost fish to mammals (Reichert et al., 2019; Xu et al., 2026). The neuropeptide orexin stabilizes wakefulness (Figure 1A) via excitatory drive to wake-promoting neurons, and the loss of orexin results in the collapse of stable state boundaries across species (Yokogawa et al., 2007; Soya and Sakurai, 2020), and underlies the sleep disorder narcolepsy. The fundamental circuit logic appears to be conserved across all examined vertebrate classes, though the relative weighting of individual nodes and their connectivity differ between diurnal and nocturnal species.

Despite the lack of anatomical homologs of the mammalian hypothalamus or brainstem in invertebrates, an analogous framework of sleep-wake circuits has been characterized. In *Drosophila melanogaster* sleep is regulated primarily by a diffuse network of neurons, specifically the dorsal fan-shaped body (dFB) and the ellipsoid body (EB) of the central complex (Figure 1B). The dFB contains sleep-promoting cholinergic and glutamatergic neurons that gate downstream locomotion and sensory circuits. These neurons also accumulate homeostatic sleep pressure in proportion to prior waking duration (Donlea et al., 2014; Jones et al., 2025). Upstream wake-promoting dopaminergic neurons modulate dFB activity to suppress sleep drive and promote arousal, paralleling the ARAS in vertebrates (Donlea et al., 2014). In *Caenorhabditis elegans*, sleep is reduced to the simplest circuit form through a single neuron, RIS (Figure 1C). RIS releases GABA and the RFamide neuropeptide FLP-11, and is activated at the onset of sleep to inhibit motor and sensory circuits, in part through the silencing of wake-promoting AIY neurons (Turek et al., 2016; Steuer Costa et al., 2019; Park et al., 2026). AIY wake-promoting neurons release a somatostatin/allatostatin ortholog NLP-99, which might in turn silence the activity of the RIS neuron (Park et al., 2026). Although the invertebrate anatomical structures are non-homologous, the circuit-level logic remains: a bi-stable circuit for arousal-sleep switching is necessary.

## The duality of sleep states–REM sleep across species

In addition to the Non-Rapid Eye Movement (NREM) sleep-promoting VLPO, the circuit controlling Rapid Eye Movement (REM) sleep is also conserved in mammals (Peever and Fuller, 2016), with the necessary core structures located in the brainstem. Glutamatergic neurons of the sublaterodorsal tegmental (SLD) nucleus are active during REM sleep and send descending projections to inhibitory neurons of the ventral medulla and spinal cord, which silence skeletal motoneurons and ascending projections (Fraigne et al., 2015). The state of REM sleep appears conserved across vertebrates and invertebrates; however, whether the circuit mechanisms are conserved remains to be elucidated (Yamazaki et al., 2020; Tainton-Heap et al., 2021; Blumberg et al., 2026). Phasic motor activity is likely a conserved feature of REM sleep: mammals exhibit rapid eye movement and limb twitches, octopi (Pophale et al., 2023) and cuttlefish (Frank et al., 2012) have irregular limb movements, and jumping spiders have rapid retinal movement and limb twitches (Rößler et al., 2022). Blumberg and colleagues have proposed that the evolutionary persistence of this sleep phasic activity is crucial for the development and maintenance of the sensorimotor system. One conserved structure that might underlie this phasic activity seen in REM/Active sleep is the red nucleus, a part of the rubrospinal tract (Blumberg et al., 2026).

## Sleep-wake structural modulation in diurnal vs. nocturnal species

**FIGURE 2:**
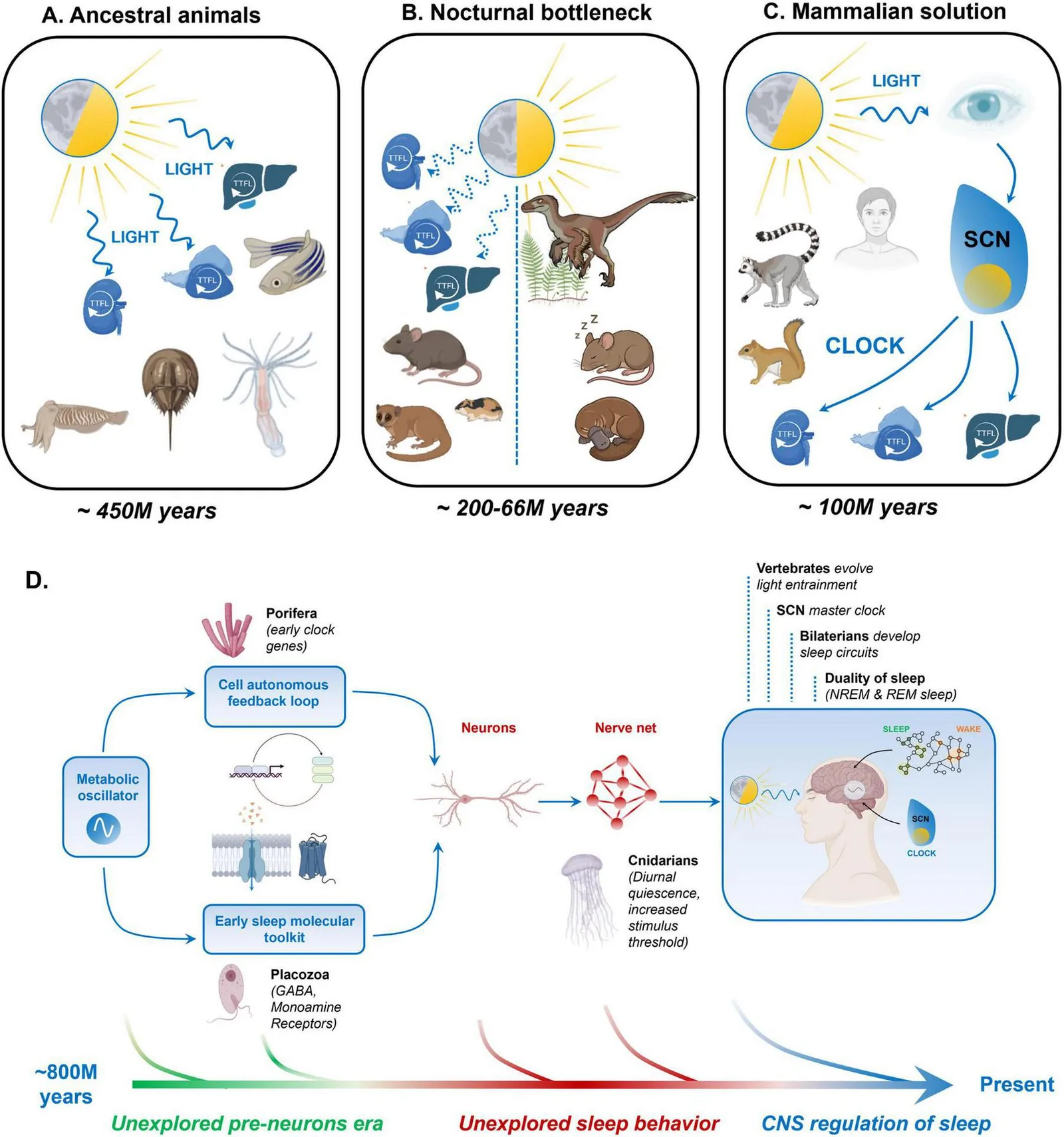
Progressive layering of sleep-regulatory mechanisms during animal evolution. **(A)** Light from the environment entrained organs directly through the activity of the TTFL present in light sensitive peripheral organs of ancestral animals (∼450 MYA). **(B)** During the Mesozoic period, mammals avoided daytime predation by adopting a nocturnal phenotype (right, ∼200–66 MYA). Due to the prolonged nocturnal period mammals lost the ability for peripheral light entrainment (left). **(C)** In mammals, the circadian rhythm was centralized to the SCN: light perceived by the eye and transmitted via the retinohypothalamic tract to the SCN, which coordinates physiological processes of the peripheral organs. **(D)** This schematic timeline illustrates the addition of molecular, cellular and circuit regulation of sleep behaviors. Ancient metabolic oscillators, such as PRX, were present in the first animals. Additional cell autonomous transcriptional feedback loops of clock genes orthologs are present in Porifera (except *period*), while molecules highly implicated in regulating sleep behaviors including GABA and monoamine receptors are present in both Porifera and Placozoa before the emergence of neurons. The evolution of neurons and their organization into nerve nets added another layer of regulation, coinciding with the earliest sleep behaviors observed in Cnidaria. In bilaterians, a centralized nervous system evolved with dedicated neurons and complex circuits to regulate sleep-wake behaviors. Finally, in vertebrates the SCN became the master timekeeper and NREM and REM sleep architecture evolved. The colored timeline highlights that much of the evolutionary history remains unexplored, particularly in animals lacking a CNS. Created in part with BioRender. Pressey, J. (2026) https://BioRender.com/6yskwdb, and in part with FigureLabs (https://chat.figurelabs.ai/verify/FL-PUB-202607 31-YKU8BO).

Molecular evidence suggests that ancestral animals were diurnal, and environmental light directly entrains the TTFL present in organs and tissues (Plautz et al., 1997; Whitmore et al., 1998, 2000). Mammals likely evolved a nocturnal phenotype during the Mesozoic era to avoid diurnal predation, often referred to as the “nocturnal bottleneck” (Walls, 1942; Figures 2A–C). Menaker et al. (1997) further hypothesized that mammals lacked extraretinal entrainment, a theory that was later validated using genomic and chronobiological evidence (Whitmore et al., 2000; Gerkema et al., 2013). Consequently, the suprachiasmatic nucleus (SCN) became the dominant brain region responsible for synchronizing the TTFL to the environment (Moore and Eichler, 1972; Stephan and Zucker, 1972) by receiving photic information via the retinohypothalamic tract (RHT; Moore and Lenn, 1972). This model is well established in mammals, and species-specific homologous structures have been identified in amphibians (Tuinhof et al., 1994), reptiles (Janik et al., 1990), birds (Cassone and Moore, 1987), and teleost fish (Burrill and Easter, 1994), but whether the SCN is the dominant circadian pacemaker in teleosts remains debated. Invertebrates express an analogous structure: the small ventrolateral neurons (sLNvs) of the circadian pacemaker circuit coordinate timing of sleep in *Drosophila* (Hsu and Sehgal, 2018).

The long-standing observation that the timing of SCN function is the same in both diurnal and nocturnal species suggests that the divergence arises from a downstream mechanism that links the SCN to the sleep-wake system. Comparative studies in diurnal Nile grass rats (*Arvicanthis niloticus*) and nocturnal laboratory rats (*Rattus norvegicus*) have demonstrated that the SCN molecular clock is identical between the two species, with peak PER1 expression at midday (Ramanathan et al., 2006). In contrast, examining PER expression in the s-LNvs and large-lateral ventral neurons (l-LNvs) in mosquitos, revealed an antiphasic expression profile linked to the light cycle. PER expression peaked in the diurnal species *Ae. aegypti* in late night/early day, while it was maximally expressed in late day/early night in the nocturnal species *An. Coluzzi* (Baik et al., 2020). While the complete circuit has not be examined in insects, evidence from rats suggests that nuclei downstream of the SCN [the paraventricular nucleus (PVN) and dorsomedial hypothalamus (DMH)] are the primary sites where chronotype-shift is determined (Nunez et al., 1999; Saper et al., 2005; Ono et al., 2020, 2024). SCN activity remains locked to the light cycle in both diurnal and nocturnal species; however, the activity of downstream PVN neurons is either silenced in nocturnal species or activated in diurnal species (Jiang et al., 2024).

Nevertheless, recent work revealed that the SCN itself is not neurophysiologically equivalent across all chronotypes. Electrophysiological recordings made from SCN neurons of the diurnal four-striped grass mouse *Rhabdomys pumilio*, showed the overall day-night pattern of firing was similar to that of nocturnal rodents (Bano-Otalora et al., 2021). Furthermore, the proportion of SCN neurons responding to RHT stimulation with inhibition, rather than excitation, was higher in *R. pumilio* than in nocturnal mice (Schoonderwoerd et al., 2022). This suggests that diurnal mammals could receive different light-mediated inputs from the RHT to the SCN, indicating that the SCN itself could contribute to the chronotype-shift in different species. While the underlying locus of regulation remains unresolved in mammals, diverse species that express primitive behaviors continue to synchronize their internal physiology to their environments using evolutionarily older regulatory mechanisms.

## Evolutionary origins of sleep

Recalling Freiberg’s temporal niche hypothesis, sleep may have evolved before a CNS existed. Highly conserved cellular regulatory circuits evolved long before neural circuits, and here we discuss four lines of evidence supporting the conclusion that sleep behavior gated by the circadian clock evolved prior to a CNS.

## Layering circadian oscillators

When atmospheric oxygen began increasing ∼2.5 billion years ago, cells progressively evolved the capability for aerobic respiration (Lyons et al., 2014). This process produces oscillations in toxic reactive oxygen species (ROS), which are neutralized by antioxidants. These rhythms occur over a ∼24-h period and appear in all aerobic organisms regardless of chronotype, with the most highly conserved antioxidant being perioxiredoxin protein (PRX), which evolved in all three domains of life (O’Neill and Reddy, 2011; Edgar et al., 2012). This metabolic oscillator does not require protein transcription, as opposed to the TTFL described above, and is even observed in red blood cells (Hoyle and O’Neill, 2015). Eukaryotes evolved additional lineage-specific layers of circadian oscillators acting at the transcription and post-translational levels (Edgar et al., 2012). Molecular oscillations are cell-autonomous drivers of circadian rhythms that optimize an organism’s physiology to match their temporal niche. These ancient processes were occurring prior to the advent of the neuron, making them an ideal mechanism to restrict animal behavior to a temporal niche.

## Double-stranded (DS) DNA breaks

All organisms accumulate DS DNA breaks as a consequence of cellular metabolism, and it is widely accepted that the timing of repair is regulated by the circadian clock (Pregueiro et al., 2006; Gamsby et al., 2009; Aguillon et al., 2026). Interestingly, the detection of DS DNA breaks signals to the TTFL to modulate the timing of the clock. In both *Neurospora crassa* and mammals DS DNA breaks activate checkpoint kinase orthologs (PRD-4 and checkpoint kinase 2, respectively), which trigger degradation of their respective clock proteins, Frequency (FRQ) and PER. This process advances the circadian clock and regulates the timing of DNA repair (Pregueiro et al., 2006; Gamsby et al., 2009). The accumulation of DS DNA breaks correlates with increased homeostatic sleep pressure in both diurnal (*Cassiopea andromeda)* and nocturnal (*Nematostella vectensis*) species of Cnidarians (Aguillon et al., 2026) and zebrafish (Zada et al., 2019, 2021), which is then released when sleep occurs and the DNA damage is repaired. Whether DNA breaks created a selective pressure favoring the evolution of sleep, or if this is simply a by-product of being in a quiescent state, remains to be elucidated. This ancient cellular mechanism for keeping DNA in optimal condition is intricately dependent on the circadian clock and provides strong evidence that some form of sleep behavior evolved before neurons.

## Sleep in animals without a CNS

To date, relatively few studies have reported the presence of sleep-like behaviors in Cnidarians, which are a phylum of animals that have neurons but no CNS (Leach et al., 2025). A landmark publication in 2017 examined circadian behaviors in *Cassiopea* spp., and Nath et al. (2017) demonstrated that rhythmic behavioral patterns of activity correspond to sleep. *Cassiopea* are jellyfish that live in shallow seawater and continuously pulse their bodies to supply oxygen and nutrients to their tissues (Hamlet et al., 2011; Santhanakrishnan et al., 2012). The pulsation rate was quantified over a 24-h time period and revealed a significant reduction in this rate at night (Nath et al., 2017). Additionally at night, *Cassiopea* showed increased threshold to stimuli during the periods of reduced activity, and when sleep deprived in the final 6 h of the night, they displayed homeostatic compensation of their sleep drive the following morning (Nath et al., 2017). This was the first demonstration of an animal without a CNS exhibiting a behavior that meets the criteria for true sleep. Whether this nightly quiescent period evolved independently of its symbiotic relationship with a photosynthetic zooxanthellae remains to be investigated (Verde and McCloskey, 1998). Nevertheless, *Cassiopea* reduces activity at night as it is energetically unfavorable to be active in the absence of photosynthesis, supporting Freiberg’s hypothesis. The emergence of Cnidarian nerve nets may be the foundation for neural control of sleep, and further studies should take advantage of the recent development of genetic tools available to investigate invertebrate models (Yamamoto and Yuste, 2020; Hanson et al., 2024; Leach et al., 2025).

## The evolution of the sleep molecular toolkit

With evidence showing that *Cassiopea* display sleep behavior, a major open question is whether the molecular toolkit regulating this behavior is conserved, which would suggest sleep is an ancient process that predates neural circuits. The model organism *Hydra* has a diffuse nerve net but no CNS and expresses neurosecretory molecules identical to those that regulate mammalian sleep circuits. *Hydra* showed increased bouts and longer periods of quiescence when treated with the highly conserved molecules melatonin, GABA and dopamine (Peres et al., 2014; Kanaya et al., 2020), which is consistent with findings from invertebrates (Kanaya et al., 2020). Interestingly, the effect of dopamine in *Hydra* was opposite to the arousing effects reported in mammals (Kanaya et al., 2020). When researchers sleep-deprived *Hydra* and assayed changes in sleep regulatory gene expression, multiple genes were altered. The strongest homologs include the sleep-promoting cyclic guanosine monophosphate (cGMP)-dependent protein kinase 1 (PRKG1) that is also expressed in *C. elegans*, *Drosophila* and mammals (Kanaya et al., 2020), and the sleep-promoting voltage-gated potassium channel *Shaker* in *Drosophila* (Cirelli et al., 2005). When Kanaya et al. (2020) knocked down homologous genes expressed in *Drosophila*, waking activity was significantly reduced demonstrating conservation of the molecular mechanism regulating sleep in both *Hydra* and *Drosophila*, and suggest deep homology of the molecular toolkit (Shubin et al., 2009).

Indeed, whole genome sequencing of the Placozoan *Trichoplax adhaerens* revealed the presence of genes associated with neurotransmission, vesicle release machinery, and transcription factors that are potentially related to the vertebrate TTFL (Kanaya et al., 2020). Moreover, neurotransmitters and monoamines are found in all domains of life, and functionally regulate behaviors in both Placozoans (Srivastava et al., 2008; Colgren and Burkhardt, 2022, 2026; Nikitin et al., 2023) and Porifera (Ellwanger and Nickel, 2006; Kravchuk et al., 2025), animals lacking neurons. While sleep behaviors have not been explicitly examined in these organisms, the presence of core regulatory elements suggests these molecules may constitute a Character Identity Network (ChIN) (Wagner, 2007; McCune and Schimenti, 2012). If this hypothesis is confirmed, these regulatory elements may define a sleep ChIN that preceded neural or TTFL-based regulation. The circadian clock may have been subsequently recruited to restrict the timing of the behavior to optimize physiology to the temporal niche of the organism. Together this evidence demonstrates that the essential molecular toolkit was present in evolutionarily old animals and was likely expanded and adapted for the regulation of sleep as organisms became more complex and specialized within a temporal niche.

## Discussion

Here we reviewed evidence from across diverse phyla for how the ancient, pre-neural origins of sleep behaviors were built upon cell-autonomous molecular feedback loops. Since the three domains of life evolved, metabolic oscillators have been keeping time suggesting the presence of deeply homologous genes inherited from ancestral organisms (Shubin et al., 2009). The addition of lineage-specific TTFL mechanisms in progressively complex organisms allowed for the restriction of physiological processes to one temporal niche. Adding to the TTFL, it appears the neural signaling molecules present in Placozoans and sponges were co-opted to fine-tune the timing of specific behaviors. A substantial advancement was the organization of individual neurons into a nerve net allowing for whole body coordination and was the basis for the evolution of highly complex sleep circuits (Figure 2D).

Sleep states in vertebrates are commonly quantified using electromyogram (EMG) and electroencephalogram (EEG) recordings, which is technically challenging in basal invertebrates resulting in many intriguing questions that are currently difficult to address. For example, do sponges, Which express neuroid secretory cells but no nerve net (Musser et al., 2021), display quiescent behaviors that meet the criteria for sleep? Why, in some species, is the circadian rhythm decoupled from sleep/quiescent behaviors? Sponges express diurnal rhythms of several orthologous clock genes with the conspicuous absence of a *Period* ortholog (Jindrich et al., 2017), and *C. elegans* sleeps despite lacking a circadian rhythm (Raizen et al., 2008). Finally, what is the evolutionary purpose of energetically expensive REM sleep activity (Mishra and Colgin, 2019) during the quiescent period? The subdivision of sleep architecture into NREM and REM sleep states complicates the element of energy conservation proposed by Freiberg and remains an open question.

As more research examining model organisms is published, we will continue to evaluate the merit of Freiberg’s hypothesis and push the boundaries of where sleep behaviors are documented. Until these critical gaps are filled however, the question remains: Are neural circuits required for sleep?
